# [(*Z*)-*O*-Methyl-*N*-propyl­thio­carbamato-κ*S*](triphenyl­phosphine-κ*P*)gold(I)

**DOI:** 10.1107/S1600536809046856

**Published:** 2009-11-11

**Authors:** Primjira P. Tadbuppa, Edward R. T. Tiekink

**Affiliations:** a Department of Chemistry, National University of Singapore, Singapore 117543; b Department of Chemistry, University of Malaya, 50603 Kuala Lumpur, Malaysia

## Abstract

In the title compound, [Au(C_5_H_10_NOS)(C_18_H_15_P)], the Au^I^ atom is linearly coordinated within an *S*,*P*-donor set with distortion from an ideal linear geometry [S—Au—P = 176.71 (6)°] due to an intra­molecular Au⋯O contact [2.943 (4) Å]. In the crystal structure, centrosymmetrically related mol­ecules associate *via* C—H⋯O inter­actions.

## Related literature

For structural systematics and luminescence properties of phosphinegold(I) carbonimidothio­ates, see: Ho *et al.* (2006 ▶); Ho & Tiekink (2007 ▶); Kuan *et al.* (2008 ▶). For the synthesis, see Hall *et al.* (1993 ▶).
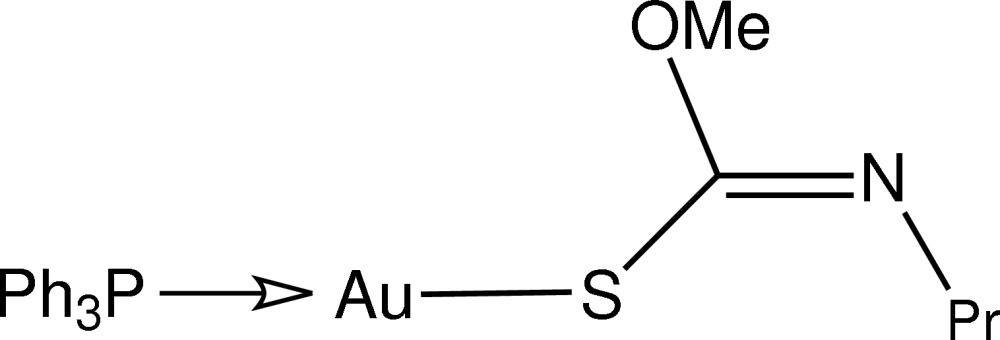


## Experimental

### 

#### Crystal data

[Au(C_5_H_10_NOS)(C_18_H_15_P)]
*M* *_r_* = 591.44
Monoclinic, 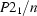

*a* = 13.9852 (16) Å
*b* = 11.1592 (13) Å
*c* = 15.0975 (17) Å
β = 107.605 (2)°
*V* = 2245.8 (4) Å^3^
*Z* = 4
Mo *K*α radiation
μ = 6.73 mm^−1^
*T* = 223 K
0.26 × 0.13 × 0.01 mm

#### Data collection

Bruker SMART CCD diffractometer
Absorption correction: multi-scan (*SADABS*; Bruker, 2000 ▶) *T*_min_ = 0.376, *T*_max_ = 1
15397 measured reflections
5165 independent reflections
4702 reflections with *I* > 2σ(*I*)
*R*_int_ = 0.047

#### Refinement

*R*[*F* ^2^ > 2σ(*F* ^2^)] = 0.045
*wR*(*F* ^2^) = 0.106
*S* = 1.20
5165 reflections
254 parameters
H-atom parameters constrained
Δρ_max_ = 1.41 e Å^−3^
Δρ_min_ = −2.23 e Å^−3^

### 

Data collection: *SMART* (Bruker, 2000 ▶); cell refinement: *SAINT* (Bruker, 2000 ▶); data reduction: *SAINT*; program(s) used to solve structure: *PATTY* in *DIRDIF92* (Beurskens *et al.*, 1992 ▶); program(s) used to refine structure: *SHELXL97* (Sheldrick, 2008 ▶); molecular graphics: *DIAMOND* (Brandenburg, 2006 ▶); software used to prepare material for publication: *SHELXL97*.

## Supplementary Material

Crystal structure: contains datablocks global, I. DOI: 10.1107/S1600536809046856/hg2583sup1.cif

Structure factors: contains datablocks I. DOI: 10.1107/S1600536809046856/hg2583Isup2.hkl

Additional supplementary materials:  crystallographic information; 3D view; checkCIF report

## Figures and Tables

**Table 1:** Selected bond lengths (Å)

Au—S1	2.3089 (16)
Au—P1	2.2557 (16)

**Table 2:** Hydrogen-bond geometry (Å, °)

*D*—H⋯*A*	*D*—H	H⋯*A*	*D*⋯*A*	*D*—H⋯*A*
C14—H14⋯O1^i^	0.94	2.51	3.314 (10)	143

Symmetry code: (i) 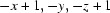.

**Fig. 1.**
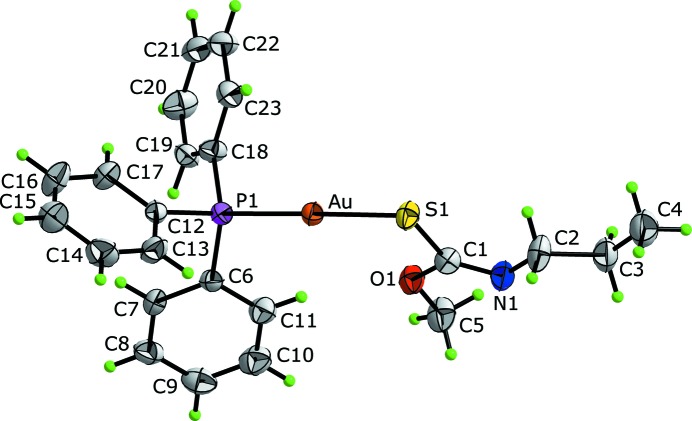
Molecular structure of (I) showing atom-labelling scheme and displacement ellipsoids at the 50% probability level.

**Fig. 2.**
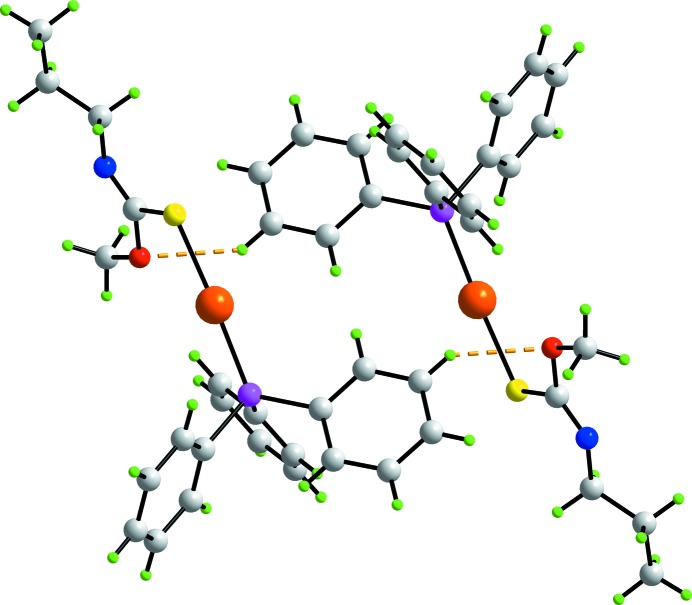
Supramolecular dimer formation in (I) mediated by C—H···O contacts (orange dashed lines). Colour code: Au, orange; S, yellow; P, pink; O, red; N, blue; C, grey; and H, green.

## supplementary crystallographic information

### Comment

The structure of the title compound, (I), was determined as a part of an on-going study of the structural systematics, including luminescence properties, of molecules related to the general formula *R*_3_PAu[SC(OR') NR''] for *R*, *R*' and *R*'' = alkyl and aryl (Ho *et al.* 2006; Ho & Tiekink, 2007; Kuan *et al.*, 2008).

The Au atom exists within the expected linear geometry defined by the S and P donor atoms. The C1–S1 and C1═N1 bond distances of 1.775 (7) and 1.265 (8) Å, respectively, confirm that the carbonimidothioate ligand is functioning as a thiolate. The small deviation from linearity about the Au atom is ascribed to the close approach of the O1 atom, Au···O1 is 2.943 (4) Å. The most prominent intermolecular interaction occurring in the crystal structure is a C–H···O contact, Table 1, which occurs between centrosymmetrically related molecules leading to a dimeric aggregate, Fig. 2.

### Experimental

Compound (I) was prepared following the standard literature procedure from the reaction of Ph_3_PAuCl and MeOC(S)N(H)Pr in the presence of base (Hall *et al.*, 1993).

### Refinement

The H atoms were geometrically placed (C—H = 0.94–0.98 Å) and refined as riding with *U*_iso_(H) = 1.2–1.5*U*_eq_(C). The maximum and minimum residual electron density peaks of 1.41 and 2.23 e Å^-3^, respectively, were located 0.82 Å and 1.59 Å from the Au and H20 atoms, respectively.

### Crystal data

**Table:**

[Au(C_5_H_10_NOS)(C_18_H_15_P)]	*F*(000) = 1152
*M**_r_* = 591.44	*D*_x_ = 1.749 Mg m^−^^3^
Monoclinic, *P*2_1_/*n*	Mo *K*α radiation, λ = 0.71069 Å
Hall symbol: -P 2yn	Cell parameters from 953 reflections
*a* = 13.9852 (16) Å	θ = 2.3–29.6°
*b* = 11.1592 (13) Å	µ = 6.73 mm^−^^1^
*c* = 15.0975 (17) Å	*T* = 223 K
β = 107.605 (2)°	Plate, colourless
*V* = 2245.8 (4) Å^3^	0.26 × 0.13 × 0.01 mm
*Z* = 4	

### Data collection

**Table:**

Bruker SMART CCD diffractometer	5165 independent reflections
Radiation source: fine-focus sealed tube	4702 reflections with *I* > 2σ(*I*)
graphite	*R*_int_ = 0.047
ω scans	θ_max_ = 27.5°, θ_min_ = 1.7°
Absorption correction: multi-scan (*SADABS*; Bruker, 2000)	*h* = −18→18
*T*_min_ = 0.376, *T*_max_ = 1	*k* = −14→8
15397 measured reflections	*l* = −19→17

### Refinement

**Table:**

Refinement on *F*^2^	Primary atom site location: structure-invariant direct methods
Least-squares matrix: full	Secondary atom site location: difference Fourier map
*R*[*F*^2^ > 2σ(*F*^2^)] = 0.045	Hydrogen site location: inferred from neighbouring sites
*wR*(*F*^2^) = 0.106	H-atom parameters constrained
*S* = 1.20	*w* = 1/[σ^2^(*F*_o_^2^) + (0.0416*P*)^2^ + 1.6273*P*] where *P* = (*F*_o_^2^ + 2*F*_c_^2^)/3
5165 reflections	(Δ/σ)_max_ = 0.001
254 parameters	Δρ_max_ = 1.41 e Å^−^^3^
0 restraints	Δρ_min_ = −2.23 e Å^−^^3^

### Special details

**Table:**

Geometry. All s.u.'s (except the s.u. in the dihedral angle between two l.s. planes) are estimated using the full covariance matrix. The cell s.u.'s are taken into account individually in the estimation of s.u.'s in distances, angles and torsion angles; correlations between s.u.'s in cell parameters are only used when they are defined by crystal symmetry. An approximate (isotropic) treatment of cell s.u.'s is used for estimating s.u.'s involving l.s. planes.
Refinement. Refinement of *F*^2^ against ALL reflections. The weighted *R*-factor *wR* and goodness of fit *S* are based on *F*^2^, conventional *R*-factors *R* are based on *F*, with *F* set to zero for negative *F*^2^. The threshold expression of *F*^2^ > 2σ(*F*^2^) is used only for calculating *R*-factors(gt) *etc*. and is not relevant to the choice of reflections for refinement. *R*-factors based on *F*^2^ are statistically about twice as large as those based on *F*, and *R*- factors based on ALL data will be even larger.

### Fractional atomic coordinates and isotropic or equivalent isotropic displacement parameters (Å^2^)

**Table:**

	*x*	*y*	*z*	*U*_iso_*/*U*_eq_	
Au	0.260547 (17)	0.07193 (2)	0.417669 (15)	0.03002 (9)	
S1	0.15590 (13)	−0.09274 (15)	0.39447 (11)	0.0358 (4)	
P1	0.35886 (12)	0.23644 (14)	0.44661 (10)	0.0289 (3)	
O1	0.1670 (3)	−0.0064 (4)	0.5583 (3)	0.0388 (10)	
N1	0.0665 (4)	−0.1720 (5)	0.5213 (4)	0.0413 (13)	
C1	0.1230 (5)	−0.0969 (6)	0.4992 (5)	0.0361 (14)	
C2	0.0194 (6)	−0.2655 (7)	0.4551 (6)	0.0488 (18)	
H2A	0.0678	−0.3301	0.4584	0.059*	
H2B	0.0002	−0.2325	0.3920	0.059*	
C3	−0.0724 (6)	−0.3162 (7)	0.4743 (6)	0.0512 (19)	
H3A	−0.1191	−0.2508	0.4744	0.061*	
H3B	−0.0526	−0.3530	0.5361	0.061*	
C4	−0.1253 (7)	−0.4091 (8)	0.4026 (7)	0.065 (2)	
H4A	−0.1845	−0.4379	0.4167	0.097*	
H4B	−0.0803	−0.4756	0.4040	0.097*	
H4C	−0.1449	−0.3730	0.3412	0.097*	
C5	0.1410 (6)	−0.0002 (8)	0.6435 (5)	0.0499 (19)	
H5A	0.0687	−0.0055	0.6297	0.075*	
H5B	0.1644	0.0752	0.6745	0.075*	
H5C	0.1724	−0.0661	0.6837	0.075*	
C6	0.4147 (4)	0.2585 (5)	0.5707 (4)	0.0285 (12)	
C7	0.5023 (5)	0.3241 (6)	0.6067 (4)	0.0360 (14)	
H7	0.5353	0.3561	0.5662	0.043*	
C8	0.5413 (5)	0.3427 (6)	0.7009 (5)	0.0427 (16)	
H8	0.6009	0.3867	0.7246	0.051*	
C9	0.4926 (6)	0.2962 (7)	0.7608 (5)	0.0470 (17)	
H9	0.5187	0.3096	0.8251	0.056*	
C10	0.4067 (6)	0.2309 (7)	0.7263 (5)	0.0507 (19)	
H10	0.3744	0.1991	0.7674	0.061*	
C11	0.3664 (5)	0.2108 (7)	0.6318 (4)	0.0412 (15)	
H11	0.3073	0.1657	0.6088	0.049*	
C12	0.4645 (4)	0.2387 (5)	0.4006 (4)	0.0284 (12)	
C13	0.5248 (5)	0.1371 (7)	0.4103 (5)	0.0429 (16)	
H13	0.5071	0.0665	0.4355	0.052*	
C14	0.6109 (6)	0.1398 (8)	0.3828 (6)	0.053 (2)	
H14	0.6528	0.0722	0.3914	0.064*	
C15	0.6347 (6)	0.2424 (8)	0.3428 (5)	0.0516 (19)	
H15	0.6925	0.2442	0.3234	0.062*	
C16	0.5748 (6)	0.3410 (8)	0.3311 (5)	0.053 (2)	
H16	0.5918	0.4102	0.3038	0.064*	
C17	0.4894 (5)	0.3408 (6)	0.3590 (4)	0.0371 (14)	
H17	0.4483	0.4091	0.3500	0.045*	
C18	0.2886 (5)	0.3729 (6)	0.4041 (4)	0.0331 (13)	
C19	0.3025 (5)	0.4783 (7)	0.4533 (4)	0.0372 (15)	
H19	0.3493	0.4819	0.5129	0.045*	
C20	0.2475 (6)	0.5802 (7)	0.4153 (5)	0.050 (2)	
H20	0.2549	0.6512	0.4503	0.060*	
C21	0.1823 (6)	0.5762 (7)	0.3265 (5)	0.0487 (19)	
H21	0.1466	0.6453	0.3001	0.058*	
C22	0.1691 (6)	0.4714 (7)	0.2761 (5)	0.0472 (18)	
H22	0.1235	0.4688	0.2159	0.057*	
C23	0.2227 (5)	0.3702 (7)	0.3139 (5)	0.0432 (16)	
H23	0.2148	0.2993	0.2789	0.052*	

### Atomic displacement parameters (Å^2^)

**Table:**

	*U*^11^	*U*^22^	*U*^33^	*U*^12^	*U*^13^	*U*^23^
Au	0.03095 (14)	0.02962 (14)	0.02940 (14)	−0.00472 (10)	0.00897 (9)	0.00101 (9)
S1	0.0416 (9)	0.0345 (8)	0.0341 (8)	−0.0110 (7)	0.0155 (7)	−0.0047 (7)
P1	0.0293 (8)	0.0287 (8)	0.0274 (7)	−0.0022 (6)	0.0067 (6)	0.0020 (6)
O1	0.039 (3)	0.046 (3)	0.036 (2)	−0.004 (2)	0.019 (2)	−0.003 (2)
N1	0.041 (3)	0.044 (3)	0.043 (3)	−0.010 (3)	0.018 (3)	0.000 (3)
C1	0.036 (3)	0.036 (3)	0.036 (3)	−0.001 (3)	0.011 (3)	0.002 (3)
C2	0.052 (5)	0.043 (4)	0.060 (5)	−0.017 (3)	0.030 (4)	−0.005 (4)
C3	0.040 (4)	0.052 (5)	0.066 (5)	−0.009 (3)	0.022 (4)	0.003 (4)
C4	0.062 (6)	0.065 (6)	0.068 (6)	−0.022 (5)	0.021 (4)	−0.002 (5)
C5	0.054 (5)	0.061 (5)	0.042 (4)	−0.011 (4)	0.027 (3)	−0.013 (4)
C6	0.029 (3)	0.031 (3)	0.025 (3)	0.002 (2)	0.007 (2)	0.002 (2)
C7	0.036 (3)	0.035 (3)	0.037 (3)	−0.006 (3)	0.012 (3)	0.006 (3)
C8	0.044 (4)	0.040 (4)	0.035 (3)	−0.001 (3)	−0.001 (3)	−0.002 (3)
C9	0.050 (4)	0.054 (5)	0.034 (3)	0.009 (4)	0.008 (3)	−0.006 (3)
C10	0.055 (5)	0.059 (5)	0.042 (4)	0.005 (4)	0.021 (3)	0.004 (4)
C11	0.039 (4)	0.051 (4)	0.036 (3)	0.002 (3)	0.015 (3)	0.002 (3)
C12	0.026 (3)	0.035 (3)	0.025 (3)	−0.005 (2)	0.008 (2)	−0.001 (2)
C13	0.044 (4)	0.038 (4)	0.050 (4)	0.007 (3)	0.019 (3)	0.014 (3)
C14	0.043 (4)	0.058 (5)	0.060 (5)	0.017 (4)	0.017 (4)	0.011 (4)
C15	0.049 (4)	0.071 (6)	0.041 (4)	0.001 (4)	0.023 (3)	−0.003 (4)
C16	0.064 (5)	0.051 (5)	0.058 (5)	−0.004 (4)	0.038 (4)	0.008 (4)
C17	0.047 (4)	0.031 (3)	0.039 (3)	0.002 (3)	0.021 (3)	0.005 (3)
C18	0.037 (3)	0.026 (3)	0.033 (3)	0.004 (3)	0.005 (3)	−0.001 (3)
C19	0.025 (3)	0.050 (4)	0.034 (3)	0.000 (3)	0.004 (2)	0.002 (3)
C20	0.065 (5)	0.036 (4)	0.049 (4)	0.007 (3)	0.016 (4)	−0.002 (3)
C21	0.050 (4)	0.053 (5)	0.046 (4)	0.013 (4)	0.019 (3)	0.018 (4)
C22	0.042 (4)	0.057 (5)	0.039 (4)	0.008 (4)	0.007 (3)	0.012 (3)
C23	0.044 (4)	0.043 (4)	0.039 (4)	−0.007 (3)	0.007 (3)	0.000 (3)

### Geometric parameters (Å, °)

**Table:**

Au—S1	2.3089 (16)	C9—C10	1.367 (11)
Au—P1	2.2557 (16)	C9—H9	0.9400
S1—C1	1.775 (7)	C10—C11	1.385 (10)
P1—C12	1.813 (6)	C10—H10	0.9400
P1—C6	1.816 (6)	C11—H11	0.9400
P1—C18	1.821 (6)	C12—C13	1.394 (9)
O1—C1	1.364 (8)	C12—C17	1.396 (8)
O1—C5	1.440 (7)	C13—C14	1.387 (10)
N1—C1	1.265 (8)	C13—H13	0.9400
N1—C2	1.457 (9)	C14—C15	1.382 (11)
C2—C3	1.508 (9)	C14—H14	0.9400
C2—H2A	0.9800	C15—C16	1.361 (11)
C2—H2B	0.9800	C15—H15	0.9400
C3—C4	1.520 (11)	C16—C17	1.380 (10)
C3—H3A	0.9800	C16—H16	0.9400
C3—H3B	0.9800	C17—H17	0.9400
C4—H4A	0.9700	C18—C19	1.373 (9)
C4—H4B	0.9700	C18—C23	1.395 (9)
C4—H4C	0.9700	C19—C20	1.395 (10)
C5—H5A	0.9700	C19—H19	0.9400
C5—H5B	0.9700	C20—C21	1.375 (10)
C5—H5C	0.9700	C20—H20	0.9400
C6—C7	1.389 (8)	C21—C22	1.377 (11)
C6—C11	1.402 (9)	C21—H21	0.9400
C7—C8	1.377 (9)	C22—C23	1.380 (10)
C7—H7	0.9400	C22—H22	0.9400
C8—C9	1.388 (10)	C23—H23	0.9400
C8—H8	0.9400		
			
P1—Au—S1	176.71 (6)	C10—C9—C8	120.0 (6)
C1—S1—Au	102.0 (2)	C10—C9—H9	120.0
C12—P1—C6	104.3 (3)	C8—C9—H9	120.0
C12—P1—C18	105.6 (3)	C9—C10—C11	121.1 (7)
C6—P1—C18	105.5 (3)	C9—C10—H10	119.5
C12—P1—Au	117.2 (2)	C11—C10—H10	119.5
C6—P1—Au	110.9 (2)	C10—C11—C6	119.3 (7)
C18—P1—Au	112.3 (2)	C10—C11—H11	120.4
C1—O1—C5	115.8 (5)	C6—C11—H11	120.4
C1—N1—C2	118.9 (6)	C13—C12—C17	119.1 (6)
N1—C1—O1	120.8 (6)	C13—C12—P1	119.1 (5)
N1—C1—S1	127.1 (5)	C17—C12—P1	121.8 (5)
O1—C1—S1	112.1 (5)	C14—C13—C12	120.2 (7)
N1—C2—C3	111.8 (6)	C14—C13—H13	119.9
N1—C2—H2A	109.3	C12—C13—H13	119.9
C3—C2—H2A	109.3	C15—C14—C13	119.6 (7)
N1—C2—H2B	109.3	C15—C14—H14	120.2
C3—C2—H2B	109.3	C13—C14—H14	120.2
H2A—C2—H2B	107.9	C16—C15—C14	120.4 (7)
C2—C3—C4	112.2 (7)	C16—C15—H15	119.8
C2—C3—H3A	109.2	C14—C15—H15	119.8
C4—C3—H3A	109.2	C15—C16—C17	121.0 (7)
C2—C3—H3B	109.2	C15—C16—H16	119.5
C4—C3—H3B	109.2	C17—C16—H16	119.5
H3A—C3—H3B	107.9	C16—C17—C12	119.6 (7)
C3—C4—H4A	109.5	C16—C17—H17	120.2
C3—C4—H4B	109.5	C12—C17—H17	120.2
H4A—C4—H4B	109.5	C19—C18—C23	119.4 (6)
C3—C4—H4C	109.5	C19—C18—P1	123.6 (5)
H4A—C4—H4C	109.5	C23—C18—P1	116.9 (5)
H4B—C4—H4C	109.5	C18—C19—C20	120.4 (6)
O1—C5—H5A	109.5	C18—C19—H19	119.8
O1—C5—H5B	109.5	C20—C19—H19	119.8
H5A—C5—H5B	109.5	C21—C20—C19	119.7 (7)
O1—C5—H5C	109.5	C21—C20—H20	120.2
H5A—C5—H5C	109.5	C19—C20—H20	120.2
H5B—C5—H5C	109.5	C20—C21—C22	120.3 (7)
C7—C6—C11	119.1 (6)	C20—C21—H21	119.8
C7—C6—P1	121.8 (5)	C22—C21—H21	119.8
C11—C6—P1	119.0 (5)	C23—C22—C21	120.0 (7)
C8—C7—C6	120.6 (6)	C23—C22—H22	120.0
C8—C7—H7	119.7	C21—C22—H22	120.0
C6—C7—H7	119.7	C22—C23—C18	120.1 (7)
C7—C8—C9	119.9 (7)	C22—C23—H23	119.9
C7—C8—H8	120.0	C18—C23—H23	119.9
C9—C8—H8	120.0		
			
C2—N1—C1—O1	178.7 (6)	C6—P1—C12—C17	−99.5 (5)
C2—N1—C1—S1	−1.6 (10)	C18—P1—C12—C17	11.5 (6)
C5—O1—C1—N1	−2.7 (9)	Au—P1—C12—C17	137.5 (5)
C5—O1—C1—S1	177.6 (5)	C17—C12—C13—C14	3.0 (10)
Au—S1—C1—N1	−179.7 (6)	P1—C12—C13—C14	−174.6 (6)
Au—S1—C1—O1	0.1 (5)	C12—C13—C14—C15	−2.3 (12)
C1—N1—C2—C3	−158.4 (7)	C13—C14—C15—C16	0.7 (13)
N1—C2—C3—C4	176.9 (7)	C14—C15—C16—C17	0.1 (13)
C12—P1—C6—C7	29.9 (6)	C15—C16—C17—C12	0.7 (12)
C18—P1—C6—C7	−81.2 (6)	C13—C12—C17—C16	−2.2 (10)
Au—P1—C6—C7	156.9 (5)	P1—C12—C17—C16	175.3 (6)
C12—P1—C6—C11	−151.9 (5)	C12—P1—C18—C19	−93.4 (6)
C18—P1—C6—C11	97.1 (6)	C6—P1—C18—C19	16.7 (7)
Au—P1—C6—C11	−24.8 (6)	Au—P1—C18—C19	137.7 (5)
C11—C6—C7—C8	−0.3 (10)	C12—P1—C18—C23	81.9 (6)
P1—C6—C7—C8	177.9 (5)	C6—P1—C18—C23	−167.9 (5)
C6—C7—C8—C9	−0.4 (11)	Au—P1—C18—C23	−46.9 (6)
C7—C8—C9—C10	0.8 (11)	C23—C18—C19—C20	3.2 (10)
C8—C9—C10—C11	−0.5 (12)	P1—C18—C19—C20	178.5 (6)
C9—C10—C11—C6	−0.1 (12)	C18—C19—C20—C21	−2.9 (11)
C7—C6—C11—C10	0.5 (10)	C19—C20—C21—C22	1.9 (12)
P1—C6—C11—C10	−177.8 (6)	C20—C21—C22—C23	−1.2 (12)
C6—P1—C12—C13	78.1 (6)	C21—C22—C23—C18	1.5 (11)
C18—P1—C12—C13	−170.9 (5)	C19—C18—C23—C22	−2.6 (11)
Au—P1—C12—C13	−45.0 (6)	P1—C18—C23—C22	−178.1 (6)

### Hydrogen-bond geometry (Å, °)

**Table:**

*D*—H···*A*	*D*—H	H···*A*	*D*···*A*	*D*—H···*A*
C14—H14···O1^i^	0.94	2.51	3.314 (10)	143

Symmetry codes: (i) −*x*+1, −*y*, −*z*+1.
